# mTOR Signaling: Recent Progress

**DOI:** 10.3390/ijms25052587

**Published:** 2024-02-23

**Authors:** Antonios N. Gargalionis, Kostas A. Papavassiliou, Athanasios G. Papavassiliou

**Affiliations:** 1 Department of Biopathology, ‘Eginition’ Hospital, Medical School, National and Kapodistrian University of Athens, 11528 Athens, Greece; 2 ‘Sotiria’ Hospital, Medical School, First University Department of Respiratory Medicine, National and Kapodistrian University of Athens, 11527 Athens, Greece; konpapav@med.uoa.gr; 3 Department of Biological Chemistry, Medical School, National and Kapodistrian University of Athens, 11527 Athens, Greece

In the intricate landscape of human biology, the mechanistic target of rapamycin (mTOR) emerges as a key regulator, orchestrating a vast array of processes in health and disease. mTOR is an evolutionarily conserved serine/threonine kinase that participates in multielement complexes with varying functional roles, depending on the established intermolecular associations and upstream cues [1]. The mTORC1 complex contains Raptor as a major component and responds to environmental/cellular stresses such as DNA damage and reactive oxygen species (ROS), the degree of the abundance of amino acids and growth factors, and energy from glucose and other sources [2,3]. mTORC1 mediates translation and protein synthesis, autophagy, lysosomal and mitochondrial biogenesis, lipid and nucleotide synthesis, mainly through upstream receptor tyrosine kinase (RTK) signaling. RTK concomitant kinase cascades include cytokine, insulin-like growth factor-1 (IGF-1), Wnt, phosphoinositide 3-kinase (PI3K)/AKT, mitogen-activated protein kinase (MAPK), and 5′ adenosine monophosphate-activated protein kinase (AMPK) pathways, most of which integrate into the tuberous sclerosis complex 1/2 (TSC1/TSC2) [4,5,6]. The mTORC2 complex is characterized by the presence of the Rictor component and regulates ion transport, cell survival, proliferation, migration, cytoskeleton remodeling, and glucose metabolism, mainly through IGF and PI3K/AKT signaling [7]. Furthermore, mTORC1 and mTORC2 are mutually regulated through diverse mTOR-associated signaling components, thus forming an intricate regulatory network that governs cellular homeostasis and disease pathogenesis [8].

The mechanisms though which mTOR specifically regulates autophagy, a cell survival process of cellular recycling, have been extensively highlighted at the intersection of cellular homeostasis for the cell to maintain intracellular balance and respond to environmental stresses. mTOR lies in the center of a complex interplay, where autophagy functions both upstream and downstream. Mechanistically, mTOR wields a dual role both by suppressing autophagy when activated by the abundance of cellular energetics, and also favoring the same process when suppressed, thereby enhancing the mechanisms that preserve cellular nutritional status [9]. This link becomes particularly interesting in cancer progression and treatment responses, since it is not clear yet whether activation or repression of autophagy is the established tumorigenic mechanism [10,11]. In certain types of malignancy, where autophagy promotes tumor progression, stemness, and drug resistance, pharmacological inhibition of autophagy is a therapeutic approach under investigation. Additionally, targeting mTOR-mediated autophagy has been proven to alleviate drug resistance, whereas drug resistance induced by mTOR suppresses autophagy and creates a favorable environment for the therapeutic exploitation of cancer metabolism [12,13].

Dysregulation of the mTOR pathway in cancer occurs due to genetic and epigenetic alterations, disturbed homeostasis of upstream regulators, and post-translational modifications [14]. During tumorigenesis, upregulation of oncogenes and downregulation of tumor-suppressors lead to hyperactivation of the mTOR signaling network in almost 30% of the neoplasms [1,15]. These alterations include mutations in the *mTOR* gene, in the *Rheb* and *TSC1/TSC2* genes, amplification of Rictor and genetic defects of the upstream *phosphatidylinositol-4,5-bisphosphate 3-kinase catalytic subunit alpha* (*PIK3CA*)*, KRAS, AKT, insulin-like growth factor receptor* (*IGFR*), and *epidermal growth factor receptor* (*EGFR*) genes [1]. Moreover, mTOR signaling is implicated in functional traits of immune cells and immune signaling from dendritic cells to T cells, thereby modulating the tumor immune microenvironment [16,17]. mTOR pathway signaling components also respond to the tumor microenvironment (TME) mechanical stresses through mechanosensitive protein molecules and facilitate traits of cancer cells [2,18]. Several members of the pathway are regulated by long noncoding RNAs (lncRNAs) and also contribute decisively to self-renewal, tumor promotion, and drug resistance [1].

mTOR is also critically involved in regulating both normal physiological processes and aberrant functions within the central nervous system. The mTOR signaling axis controls the survival and development of brain cells and processes of learning and memory, as well as neuronal and synaptic plasticity [19]. mTOR is mechanistically tightly interconnected with the TSC1/TSC2 complex; therefore, it has been found upregulated in patients with TSC—an autosomal dominant disorder caused by loss-of-function mutations of either *TSC1* or *TSC2* genes—who develop neurological manifestations, including epilepsy, neuropsychiatric disorders, autism, and brain tumors [20,21]. mTOR inhibition in TSC patients is promising against epilepsy, whereas mTORC1-associated autophagy has been correlated with neurodegenerative disorders such as Alzheimer’s disease and Parkinson’s disease [22,23,24]. mTOR signaling is also engaged in the regulation of cardiac physiology and corresponding cardiovascular maladies. These include pulmonary arterial hypertension, myocardial infarction, and atherosclerosis [14].

Several small-molecule compounds have been discovered that target mTOR-triggered pathobiologies, especially in various types of malignancy [1]. These compounds have already taken their place in the clinic, including rapalogs (rapamycin derivatives) or mTOR inhibitors, such as Nab-sirolimus against metastatic or unresectable PEComas (tumors showing perivascular epithelioid cell differentiation) and lymphangioleiomyomatosis (LAM) [25,26,27], temsirolimus against advanced renal cell carcinoma (RCC) [28], everolimus for advanced RCC, advanced breast carcinoma and neuroendocrine pancreatic, lung, and gastrointestinal tract carcinomas [29,30,31,32], and ridaforolimus, which demonstrates efficacy against bone sarcomas [33]. Due to resistance to rapalogs and mTOR inhibitors, dual PI3K and mTOR inhibitors have been developed [34]. The mTOR pathway can also be targeted using ATP-competitive mTOR inhibitors, PI3K and AKT inhibitors [1].

Investigation of mTOR in human pathobiology has provided a wide spectrum of therapeutic opportunities, but also continuous controversies. mTOR exerts a multifaceted role in physiology and associated disorders, evoking debates about efficient mTOR pharmacological targeting. Deciphering these complex mechanisms of mTOR function and anomalous activity will provide new tools to fully utilize the dynamics of mTOR inhibition.
